# Nicotine Exerts a Stronger Immunosuppressive Effect Than Its Structural Analogs and Regulates Experimental Colitis in Rats

**DOI:** 10.3390/biomedicines11030922

**Published:** 2023-03-16

**Authors:** Kohki Okada, Kano Matsuo

**Affiliations:** 1 Department of Medical Technology and Sciences, Faculty of Health Sciences, Kyoto Tachibana University, Kyoto 607-8175, Japan; 2 Graduate School of Health Sciences, Kyoto Tachibana University, Kyoto 607-8175, Japan

**Keywords:** anabasine, anatabine, α7 nicotine acetylcholine receptor, cotinine, macrophage, myosmine, nornicotine, nicotine, ulcerative colitis

## Abstract

Ulcerative colitis (UC) is an intractable disease that causes persistent colonic inflammation. Numerous studies have reported that smoking can afford clinical benefits in UC. This study aimed to elucidate whether nicotine, the main component in cigarettes, can exert pharmacological effects against experimental UC. To achieve this objective, we compared the effects of nicotine with those of structural nicotine analogs in a UC rodent model (Slc: Wistar rats, male, 9-week-old, and 220–250 g/rat). Nicotine, or a respective structural analog (nornicotine, cotinine, anabasine, myosmine, and anatabine), was administered intraperitoneally daily to rats (*n* = 6/group) exhibiting dextran sulfate sodium-induced experimental colitis. Examining the colon tissues of model rats, we compared disease severity, cytokine secretion, and α7 nicotine acetylcholine receptor (nAChR7) expression. We observed that nicotine administration induced weight loss at 2.35% in 10 days. Notably, the reduction in histological severity (score) of UC was more pronounced in rats treated with nicotine (score = 4.83, *p* = 0.042) than in untreated rats (score = 8.17). Nicotine administration increased nAChR7 expression 6.88-fold (*p* = 0.022) in inflammatory sites of the colon, mainly by suppressing the production of interleukin (IL)-1β and IL-6. Moreover, the secretion of these cytokines was suppressed in lipopolysaccharide-stimulated rat macrophages (MΦ) treated with nicotine. In conclusion, nicotine better alleviates experimental UC than the examined structural analogs by activating nAChR7 expression and suppressing proinflammatory cytokines in MΦ.

## 1. Introduction

Nicotine is one of the main components of cigarettes and is a known toxin stored in tobacco plants to ward off insect attacks [1,2]. Nicotine is a highly neurotoxic and addictive substance, and nicotine dependence among smokers is considered a major public health challenge. Nicotine ingestion damages the central and peripheral nervous systems, leading to acute and chronic intoxication and often sudden death [3]. Nicotine acts as an agonist of nicotine acetylcholine receptors (nAChRs) in skeletal muscle and brain tissues [4,5]. On binding to these receptors, nicotine promotes the secretion of neurotransmitters (dopamine, adrenaline, and beta-endorphins) [6]. Based on these properties, nicotine may exert therapeutic effects in certain diseases associated with cognitive and behavioral control issues, such as depression and Alzheimer’s disease [7,8]. Conversely, it has been reported that nicotine could aggravate gastrointestinal diseases. In particular, chronic nicotine administration can substantially reduce blood flow to the gastric mucosa while increasing gastric acid secretion, thereby aggravating gastritis and gastric ulcers [9,10,11].

Ulcerative colitis (UC) is an intractable disease that causes persistent inflammation in the colon, particularly in the rectal region [12]. A high prevalence of UC has been reported in Europe and the United States, with the number of patients increasing in Asia, South America, and Africa [13]. Abnormal activity of innate immune cells and disruption of the intestinal microbiota have been associated with the onset and worsening of UC [14] However, the precise underlying mechanisms remain unknown. Although therapeutic protocols capable of comprehensively curing UC remain elusive, immunosuppressive drugs such as tacrolimus and cyclosporine are frequently employed for therapeutic benefits [14]. In particular, suppressing the excessive macrophage (MΦ)-mediated immune response in colon tissues has been effective in UC [15]. Interestingly, numerous clinical and basic research reports have suggested that nicotine intake could alleviate UC [16,17,18]. Of note, nAChRs are abundantly expressed not only in skeletal muscle and brain but also in the colon mucosal epithelium and immune cells, and it has been strongly suggested that α7 nAChR (nAChR7), comprising homopentamers of the α7 subunit, may be associated with UC [5,19]. In contrast, smoking is reported to be a risk factor in Crohn’s disease [20], a disease with a similar pathology to UC, which further complicates the debate over whether nicotine has pharmacological effects.

The underlying mechanism through which nicotine and its receptors participate in the pathogenesis of UC remains unclear. We postulate that the unique chemical structure of nicotine exerts a pharmacological effect on UC. If this assumption is accurate, structural analogs of nicotine could exhibit similar therapeutic effects against UC; however, reports verifying their potential effects remain limited. Moreover, nicotine induces strong dependence and addictive properties, and structural analogs of nicotine could afford UC remission without inducing these disadvantages. In humans, the stress-relieving effects of smoking may lead to remission of UC, although examining the function of nicotine itself appears challenging. Therefore, it is crucial to investigate the functional roles of nicotine in animal models. We previously reported that S100A8 and its recombinant hybrid protein can attenuate the severity of dextran sulfate sodium (DSS)-induced UC [21,22]. Therefore, identifying the chemical structures of nicotine and its analogs that exert anti-inflammatory effects would contribute to the development of novel therapeutic agents for UC.

Based on the above background, we aimed to confirm the effects of nicotine and its five structural analogs on the pathogenesis of experimental UC in rats, as well as perform a detailed comparative analysis of their pharmacological effects.

## 2. Materials and Methods

### 2.1. Ethics Statement

All animal experiments complied with the ARRIVE guidelines (Animal Research: Reporting of In Vivo Experiments) and were approved by the Animal Experiment Committee of Kyoto Tachibana University (permission number: 21-08).

### 2.2. Animals

Wild-type (WT) Slc: Wistar rats (male, 9-week-old, and weighing 220–250 g/rat) were obtained from Shimizu Laboratory Supplies Co., Ltd. (Kyoto, Japan). Animals were housed for approximately one week prior to the experimentation with free access to diets (MF, Oriental Yeast Co. Ltd., Tokyo, Japan) and water. During the experiments, animals were kept in individual sawdust-lined plastic cages under controlled temperature (22 °C) and humidity (60%) conditions, with day–night cycles regulated by artificial light (12/12 h). The content and composition of experimental diets are presented in Figure S1 [23].

### 2.3. Reagents

Nicotine, nornicotine, cotinine, anatabine, and anti-β-actin mouse monoclonal antibody (anti-β-actin IgG) were obtained from FUJIFILM Wako Pure Chemical Corporation (Osaka, Japan). Myosmine, anti-nicotinic acetylcholine R alpha 7/CHRNA7 rabbit-poly (anti-nAChR7), and peroxidase (HRP)-labeled anti-rabbit IgG(H+L) goat-poly were obtained from Funakoshi Co., Ltd. (Tokyo, Japan). Anabasine was obtained from Angene International, Ltd. (Nanjing, China). Anti-mouse IgG (goat)-HRP conjugate and goat anti-rabbit IgG H&L (TRITC) were obtained from Abcam (Cambridge, UK). The PRO-PREPTM Protein Extraction Solution (Cell/Tissue) and interleukin (IL)-6 PicoKine^TM^, IL1-beta (IL-1β), and tumor necrosis factor-alpha (TNF-α) enzyme-linked immunosorbent assay (ELISA) kits were obtained from Cosmo Bio Co., Ltd. (Tokyo, Japan). The DSS salt (molecular weight: 36,000–50,000) was obtained from Wako Pure Chemical Industries, Ltd. (Tokyo, Japan). Clinical thioglycollate medium (E-MC17) was obtained from Eiken Chemical Co., Ltd. (Tokyo, Japan). *Escherichia coli*-derived lipopolysaccharide (LPS) was obtained from Sigma-Aldrich Co., LLC (Tokyo, Japan). VECTASHIELD mounting medium containing 4′,6-diamidino-2-phenylindole (DAPI) was obtained from Vector Inc. (Burlingame, CA, USA). TRIzol™ reagent, SuperScript ™ II Reverse Transcriptase, PowerUp SYBR Green Master Mix, and all primers were obtained from Thermo Fisher Scientific (Waltham, MA, USA). All other reagents were obtained from Wakenyaku Co., Ltd. (Kyoto, Japan), Nacalai Tesque Co., Ltd. (Kyoto, Japan), and Bio-Rad Laboratories Inc. (Hercules, CA, USA).

### 2.4. Protocol for Animal Experiments

**Figure 1:**
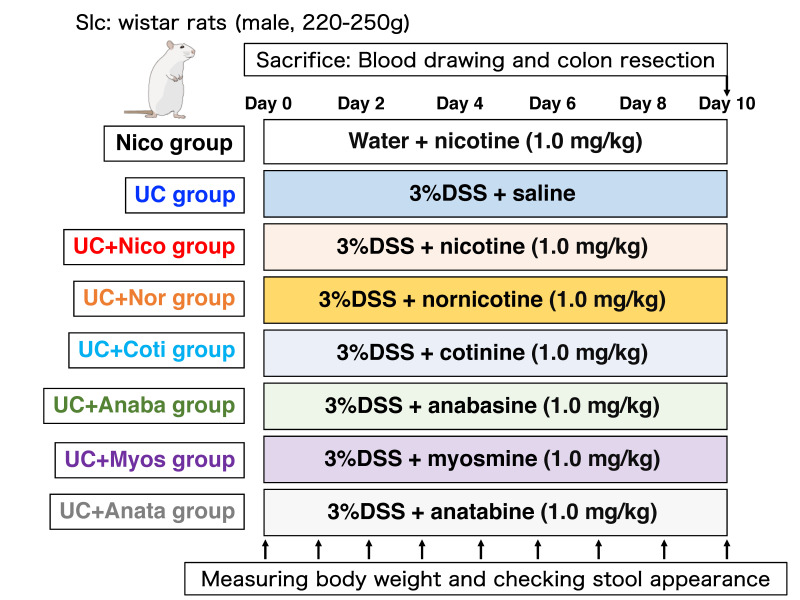
Protocol for animal experiments. Nico group, Slc: Wistar rats (wild-type [WT]) were intraperitoneally administered 1.0 mg/kg nicotine daily; in the UC group, WT rats were orally administered 3% dextran sulfate sodium (DSS), plus an equal volume of saline (1.0 mg/kg) intraperitoneally administered daily; in the UC + Nico, +Nor, +Coti, +Anaba, +Myos, and +Anata groups, WT rats were orally administered 3% DSS, plus 1.0 mg/kg nicotine, nornicotine, cotinine, anabasine, myosmine, or anatabine intraperitoneally administered daily. All rats were euthanized on day 10, followed by the harvesting of the large intestines and blood collection. Anaba, anabasine; Anata, anatabine; Coti, cotinine; Myos, myosmine; Nico, nicotine; Nor, nornicotine; and UC, ulcerative colitis.

To establish experimental UC model rats (UCR), WT rats were orally administered 3% DSS in distilled water (DW) from their water bottles for 10 days. UCR were divided into six groups (UC + Nico, UC + Nor, UC + Coti, UC + Anaba, UC + Myos, and UC + Anata), with 1.0 mg/kg of each chemical (nicotine, nornicotine, cotinine, anabasine, myosmine, and anatabine) intraperitoneally administered daily (*n* = 6). During the experimental period, the body weight of each rat was measured every morning, and disease activity index (DAI) scores were evaluated based on the criteria shown in Table 1 [24]. Considering the control group, normal WT rats were intraperitoneally administered 1.0 mg/kg nicotine daily (Nico group, *n* = 6). The dose was determined based on previous reports on the efficacy of nicotine in treating bowel inflammation [25,26]. In addition, one group of UCR was administered an equal volume of saline (UC group, *n* = 6). On day 10 of the experiment protocol, blood samples (3 mL/rat) were collected from each rat via intracardiac puncture under anesthesia. In addition, the large colon was quickly removed, and its length was measured. The specimens were fixed in 10% formalin/0.1 M phosphate buffer (pH 7.4) for histological assessments and then embedded in paraffin. The protein and mRNA levels in the residual unfixed tissue were extracted as described below. The experimental animal protocol and animal groups are summarized in Figure 1.

### 2.5. Sample Preparation for the Protein Assay

The large intestines harvested from each experimental animal were weighed independently. Further, 300 mg of each sample was incubated in 0.5 mL of PRO-PREPTM Protein Extraction Solution (Cell/Tissue) for 10 min, followed by centrifugation at 12,000 rpm for 10 min at 4 °C. The resultant supernatants were transferred into 1.5 mL polycarbonate tubes and stored at −80 °C until use.

### 2.6. Sample Preparation for the mRNA Assay

Briefly, 300 mg of each tissue was independently incubated in 1.0 mL of TRIzol™ reagent for 10 min and then centrifuged at 12,000 rpm for 15 min at 4 °C. The mRNA from each sample was extracted following the manufacturer’s protocol and treated with 8 M LiCl to avoid the influence of DSS on RNA reverse transcription. cDNA was synthesized from mRNA using SuperScript™ II reverse transcriptase, as described in the instruction manual.

### 2.7. Microscopic Examination of Harvested Rectal Tissues

We microscopically examined rectal tissues exhibiting severe inflammation in rats with DSS-induced UC. Briefly, 3 µm-thick tissue sections were prepared from the rectal tissues of all rats and stained with hematoxylin and eosin (H&E). Tissue damage was evaluated as histological (HIS) scores and assessed based on the H&E staining, and the extent of damage was scored based on established criteria. The presence and severity of ulcerative lesions, disrupted epithelial structure, damaged crypt architecture, and increased inflammatory cell infiltration were scored on a scale of 0–3 (none = 0; mild = 1; moderate = 2; and severe = 3), which were summed to provide an overall score [27]. The expression of nAChR7 in tissues and MΦ was detected by immunohistochemical staining with diaminobenzidine (DAB) and fluorescent immunochemical staining (FICS), respectively, using anti-nAChR7, as previously described [21]. Microscopic images were obtained using a BIOREVO BZ-9000 microscope (Keyence Co., Ltd., Osaka, Japan).

### 2.8. Western Blotting

The proteins in each fraction were separated by sodium dodecyl sulfate-polyacrylamide gel electrophoresis (SDS-PAGE) in the presence of 2-mercaptoethanol, as previously described [28]. The concentration of all polyacrylamide gels was 12.5%. After SDS-PAGE, proteins were transferred to nitrocellulose membranes using Trans-Blot Turbo (Bio-Rad Laboratories, Inc.). After blocking with Blocking One (Nacalai Tesque Co., Ltd.), membranes were incubated at 4 °C for 1 h with 2 μg/mL anti-nAChR7 or anti-β-actin IgG. The membranes were then washed thrice for 5 min with 10 mM Tris-HCl buffer (pH 7.4) and 0.9% NaCl (buffer A), twice with buffer A/0.1% Tween 20, and once with buffer A before incubation with 2 μg/mL HRP-labeled anti-rabbit IgG(H+L) goat poly or anti-mouse IgG (goat)-HRP conjugate at room temperature for 1 h. After the membranes were washed, antibody-bound proteins were detected using a Chemi-DocTM XRS Plus Imaging System and Clarity Western ECL substrate (Bio-Rad Laboratories, Inc.). The original Western blotting data are presented in Figure S2.

### 2.9. ELISA

IL-6, IL-1β, and TNF-α levels in each sample were measured using respective ELISA kits, following the manufacturer’s instructions. The absorbance of the color reaction was measured at a wavelength of 450 nm using a microplate reader (Bio-Rad Laboratories, Inc.). Cytokines in the large intestine were converted to concentrations per gram of the tissue.

### 2.10. Real-Time PCR

Real-time PCR was performed using the StepOnePlus Real-Time PCR System (Thermo Fisher Scientific), as previously described [21]. The primers used were as follows: nAChR7 forward, 5′-ACAATACTTCGCCAGCACCA-3′, nAChR7 reverse, 5′-AAACCATGCACACCAGTTCA-3′ (145 bp); IL-1β forward, 5′-CACCTCTCAAGGAGAGCACAGA-3′, IL-1β reverse, 5′-CACCTCTCAAGGAGAGCACAGA-3′ (81 bp); IL-6 forward, 5′-ATATGTTCTCAGGGAGATCTTGGAA-3′, IL-6 reverse, 5′-GTGCATCATCGCTGTTCATACA-3′ (80 bp); IL-10 forward, 5′-GCCAAGCCTTGTCAGAAATGA-3′, IL-10 reverse, 5′-TTTCTGGGCCATGGTTCCTCT-3′ (75 bp); TNF-α forward, 5′-GTGATCGGTCCCAACAAGGA-3′, TNF-α reverse, 5′-AGGGTCTGGGCCATGGAA-3′ (71 bp); transforming growth factor (TGF)-β forward, 5′-ACCTGCAAGACCATCGACATG-3′, TGF-β reverse, 5′-CGAGCCTTAGTTTGGACAGGAT-3′ (85 bp); and β-act forward, 5′-TGTGTTGTCCCTGTATGCCTCTG-3′, β-act reverse, 5′-ATAGATGGGCACATGGTGGGTG-3′ (85 bp).

### 2.11. Isolation of Peritoneal MΦ from Rats

Peritoneal MΦ were isolated from WT rats as previously described [24]. Briefly, MΦ were induced by intraperitoneal injection of sterilized 4% thioglycollate/DW (10 mL). After three days, MΦ were collected in a plastic tube (50 mL) using 50 mM of sterilized phosphate buffered solution (pH 7.4) and 0.9% NaCl (buffer B). Subsequently, the tube was centrifuged at 3500 rpm for 5 min at 4 °C, and the supernatant was discarded. The cells collected in the tube were suspended in 17 mM Tris-HCl (pH 7.2) containing 0.83% NH_4_Cl and incubated at 37 °C for 10 min to induce hemolysis of contaminating erythrocytes. After centrifugation, the supernatant was discarded, and pelleted cells were suspended in RPMI-1640 culture medium containing 10% fetal bovine serum (Biological Industries, Kibbutz Beit-Haemek, Israel; medium A). A total of 2 × 10^6^ cells were plated in each well of a 6-well plate, and an appropriate volume of medium A was added. The cells were incubated at 37 °C for 2 h under 5% CO_2_. After incubation, non-adherent cells in each well were removed by washing three times with buffer B. Adherent cells were maintained in the same medium at 37 °C in 5% CO_2_ until use.

### 2.12. Stimulation of MΦ

Briefly, MΦ were thoroughly washed with buffer B, followed by stimulation with 1 μg/mL nicotine, its structural analogs, or LPS in medium A for 1 h. After washing thrice with buffer B, the cells were subjected to FICS or collected with 0.2 mL of buffer A in a polycarbonate tube (1.5 mL). Protein and mRNA were extracted from collected cells using PRO-PREPTM Protein Extraction Solution and TRIzol reagent, respectively. The cytokine production capacity of MΦ was analyzed using ELISA and real-time PCR. The intracellular expression of nAChR7 was evaluated via Western blotting, real-time PCR, and FICS.

### 2.13. Statistical Analysis

Pairwise comparisons with the control were performed using non-parametric tests. Significant differences between groups were identified using the Mann–Whitney U test. The Shapiro–Wilk test was used to verify the normality of distribution, and all numerical data in this study were determined to be non-normally distributed. Then, the data are shown as median ± max/min. A *p*-value of <0.05 was deemed statistically significant.

## 3. Results

### 3.1. Changes in Body Weight

**Figure 2:**
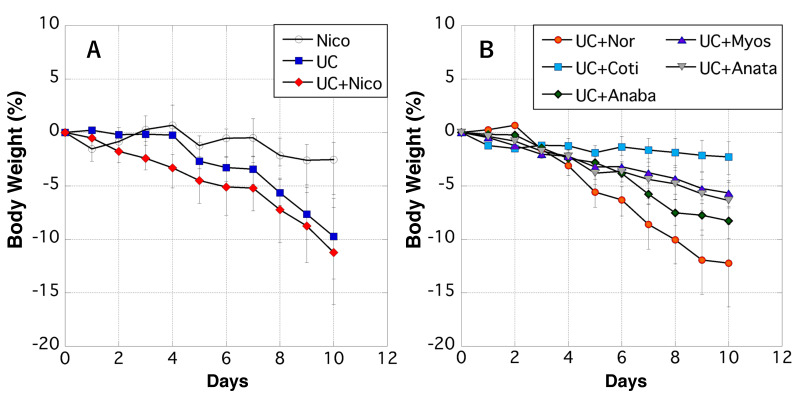
Changes in the body weight of rats. (**A**) The *y*-axis indicates the percentage fluctuation in the body weight of the Nico (white circle), UC (blue square), and UC + Nico (red diamond) groups after initiating the experiment. (**B**) The *y*-axis shows the percentage fluctuation in the body weight of the UC + Nor (yellow circle), UC + Coti (light blue square), UC + Anaba (green diamond), UC + Myos (purple triangle), and UC + Anata (gray inverted triangle) groups after initiating the experiment. (**A**,**B**) The *x*-axis indicates the number of days after initiating the experiment. Data values are presented as the median ± max/min. Anaba, anabasine; Anata, anatabine; Coti, cotinine; Myos, myosmine; Nico, nicotine; Nor, nornicotine; and UC, ulcerative colitis.

During the experimental period, we measured the body weights of rats daily. The weight of rats in the Nico group gradually decreased after day 6, whereas rats in the UC group showed rapid weight loss after day 4 (Figure 2A). Rats in the UC + Nico group exhibited greater weight loss than UC group rats (Figure 2A). Conversely, rats in the UC + Nor group displayed more severe weight loss than any other examined group (Figure 2B).

### 3.2. Changes in Rat Colon Lengths following Nicotine Administration

**Figure 3:**
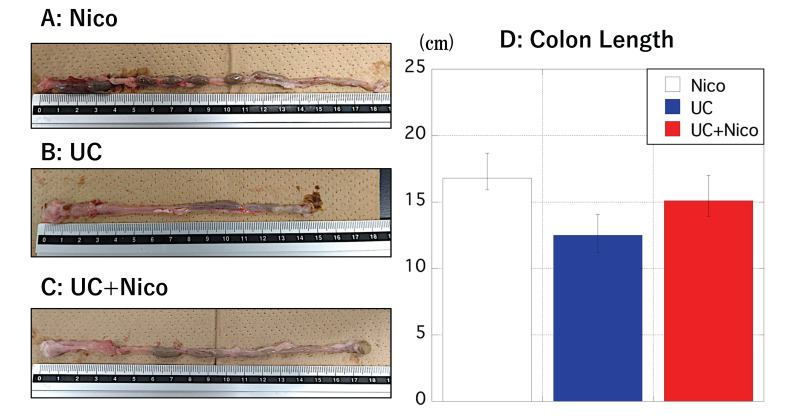
Changes in the colon length in the Nico, UC, and UC + Nico groups. (**A**–**C**) Longest colon tissue images from the Nico, UC and UC + Nico groups. (**D**) The *y*-axis indicates the colon length (in cm) of rats in Nico (white), UC (blue), and UC + Nico (red) groups after initiating the experiment. Data values are presented as the median ± max/min. Nico, nicotine; UC, ulcerative colitis.

At the end of the experimental period, rats in the UC group exhibited a visually shorter colon than those in the Nico group; however, colonic atrophy was suppressed in the UC + Nico group (Figure 3A–C). This tendency was also apparent in the mean length of the large intestine among the three groups (Figure 3D).

### 3.3. Changes in Rat Colon Lengths following Treatment with Nicotine Structural Analogs

**Figure 4:**
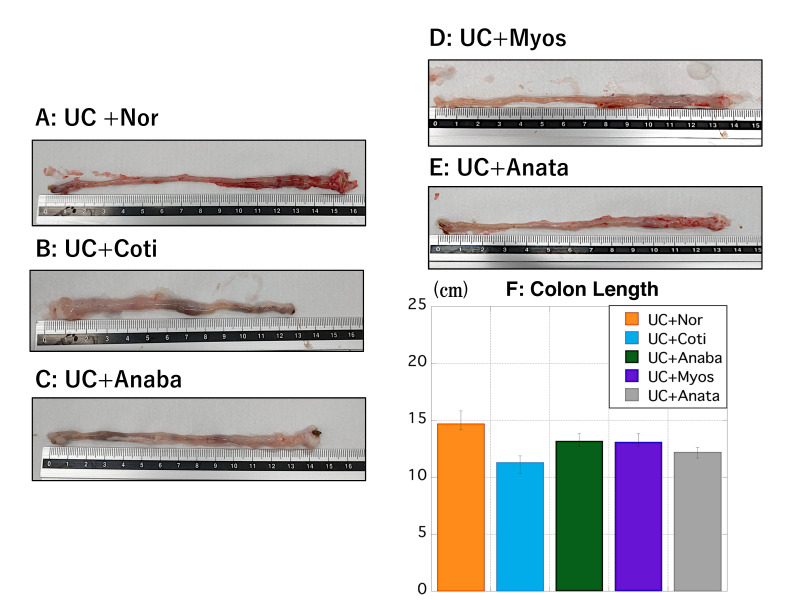
Changes in the colon length in the UC + Nico analog groups. (**A**–**E**) Images of the longest colon tissue in the UC + Nor, UC + Coti, UC + Anaba, UC + Myos, and UC + Anata groups. (**F**) The *y*-axis indicates the colon length (in cm) of rats in the UC + Nor (yellow), UC + Coti (light blue), UC + Anaba (green), UC + Myos (purple), and UC + Anata (gray) groups after initiating the experiment. Data values are presented as the median ± max/min. Nico, nicotine; UC, ulcerative colitis. Anaba, anabasine; Anata, anatabine; Coti, cotinine; Myos, myosmine; Nico, nicotine; Nor, nornicotine; and UC, ulcerative colitis.

The colon of rats in the UC + Nor group appeared visually maintained when compared with that of rats in the other four groups (Figure 4A–E). Among the five groups, the mean colon length was the longest in the UC + Nor group and the shortest in the UC + Coti group (Figure 4F).

### 3.4. Comparison of DAI Scores between the Treatment Groups

We evaluated DAI scores based on the criteria listed in Table 1, as previously described [24]. The DAI scores increased in the UC group at Day 10 as physical symptoms worsened, whereas only a moderate change was noted in the UC + Nico group (Table 2 and Figure S3). Compared to the other five groups, the UC + Nico group tended to have lower DAI scores (Table 2 and Figure S3).

### 3.5. Microscopic Observation and Evaluation of Colonic Inflammation

**Figure 5:**
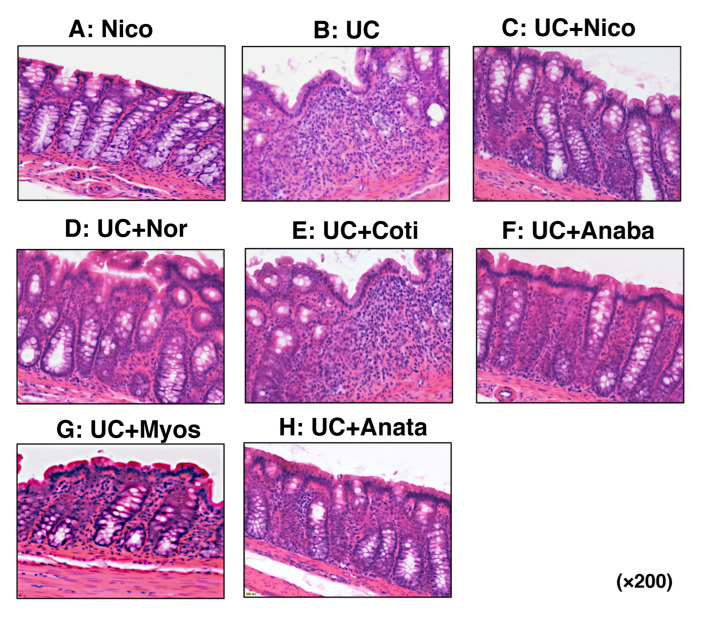
Evaluation of histological severity in colonic tissues of rats. (**A**–**H**) Microscopic images of colon tissues of from the Nico, UC, UC + Nico, UC + Nor, UC + Coti, UC + Anaba, UC + Myos, and UC + Anata groups, respectively. All microscopic images were observed using a BIOREVO BZ-9000 microscope (Keyence Co., Ltd., Osaka, Japan). High-power magnification was used for all panels (×200). Anaba, anabasine; Anata, anatabine; Coti, cotinine; DAI, disease activity index; HIS, histological; Myos, myosmine; Nico, nicotine; Nor, nornicotine; and UC, ulcerative colitis.

H&E staining was performed to visualize tissue damage and immune cells in the large intestine. Compared with colon tissue derived from the Nico group, the epithelial structure of the colon was damaged in the UC group, with numerous immune cells infiltrating the tissue (Figure 5A,B). Compared with the UC group, the UC + Nico and UC + Nor groups exhibited a preserved mucosal epithelial structure and suppressed immune cell infiltration (Figure 5C,D). Although less severe than that in the UC group, inflammatory findings were documented in harvested colons of the four other groups (Figure 5E–H). HIS scores were assessed based on previously established criteria [27]. Only the UC + Nico group showed significantly suppressed HIS scores when compared with those of the UC group (Table 3).

### 3.6. Evaluation of nAChR7 Expression in the Large Intestine of All Groups

**Figure 6:**
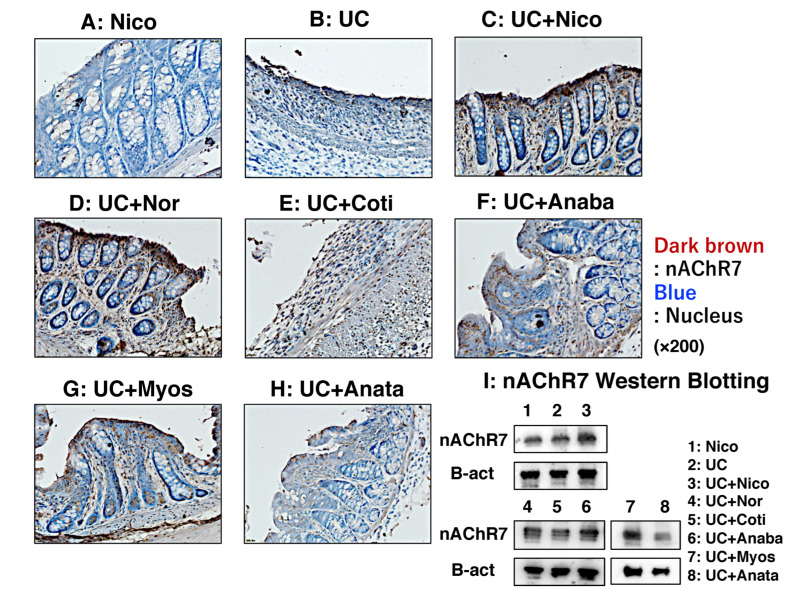
Evaluation of nAChR7 expression in colonic tissues of rats. (**A**–**H**) Results of immune staining with DAB to visualize nAChR (dark brown) in the colon tissues of the Nico, UC, UC + Nico, UC + Nor, UC + Coti, UC + Anaba, UC + Myos, and UC + Anata groups, respectively. Nuclei were stained blue with hematoxylin. All microscopic images were observed using a BIOREVO BZ-9000 microscope (Keyence Co., Ltd., Osaka, Japan). High power magnification was used for all panels (×200). (**I**) Western blotting was performed to detect nAChR7 expression in the rat colon tissues. The upper and lower panels show the expression of nAChR7 and β-actin (an internal control) proteins, respectively. Each lane 1–8 shows the results of the Nico, UC, UC + Nico, UC + Nor, UC + Coti, UC + Anaba, UC + Myos, and UC + Anata groups. Anaba, anabasine; Anata, anatabine; Coti, cotinine; Myos, myosmine; nAChR7, α7 nicotine acetylcholine receptor; Nico, nicotine; Nor, nornicotine; and UC, ulcerative colitis.

To determine nAChR7 expression in colon tissues, we performed immunohistochemical staining with DAB and Western blotting. Based on immunohistochemical staining, nAChR7 was minimally expressed in the colon of Nico and UC group rats (Figure 6A,B). In contrast, high levels of nAChR7 expression were detected in the colon of UC + Nico and UC + Nor group rats (Figure 6C,D). In addition, the other four groups exhibited some degree of nAChR7 expression (Figure 6E–H). Although we noted some extent of nAChR7 expression in all groups by Western blotting, distinct expression was noted in the UC + Nico group (Figure 6I).

### 3.7. In-Depth Investigation of nAChR7 Expression in the Large Intestines of All Groups

**Figure 7:**
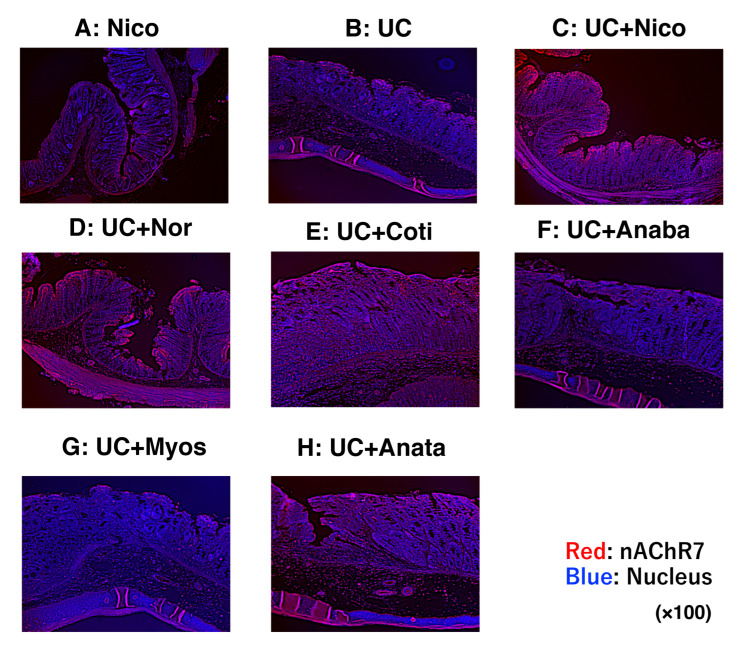
In-depth evaluation of nAChR7 expression in rat colonic tissues. (**A**–**H**) Results of FICS of nAChR (red fluorescence) in rat colonic tissues from the Nico, UC, UC + Nico, UC + Nor, UC + Coti, UC + Anaba, UC + Myos, and UC + Anata groups, respectively. DAPI-stained nuclei exhibit blue fluorescence. All microscopic images were observed using a BIOREVO BZ-9000 microscope (Keyence Co., Ltd., Osaka, Japan). High-power magnification was used for all panels (×200). Anaba, anabasine; Anata, anatabine; Coti, cotinine; FICS, fluorescent immunochemical staining; Myos, myosmine; nAChR7, α7 nicotine acetylcholine receptor; Nico, nicotine; Nor, nornicotine; and UC, ulcerative colitis.

We next performed FICS and real-time PCR to establish the expression of nAChR7 in the large intestines. FICS revealed several nAChR7-positive areas, particularly in the colonic mucosal epithelium of the UC + Nico group when compared with that in other examined groups (Figure 7A–H). In addition, the UC + Nico group exhibited the highest expression of nAChR7 mRNA in colon tissues, followed by the UC + Nor group (Table 4).

### 3.8. Cytokine Secretion in Serum and Colon

**Figure 8:**
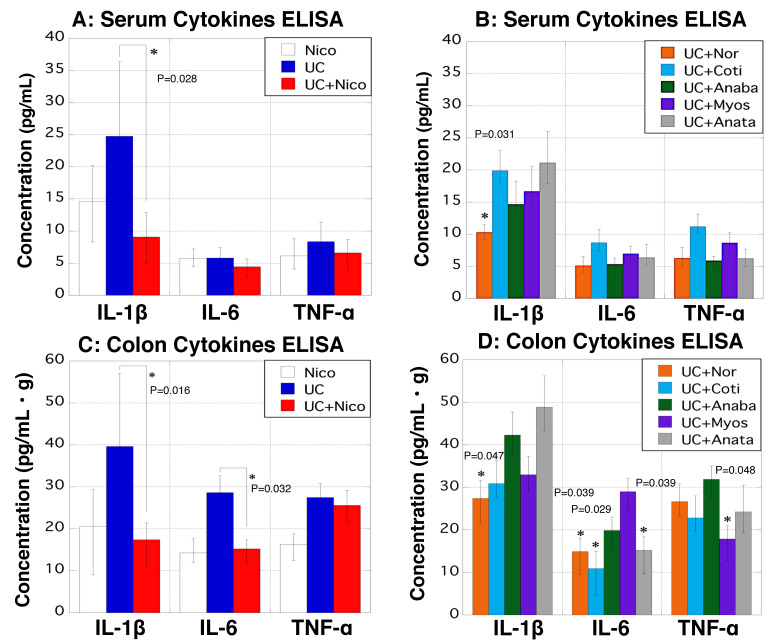
Changes in serum and colon levels of inflammatory cytokines in rats. ELISA was performed to measure cytokine levels in rat serum and colon tissues. (**A**,**B**) The *y*-axis indicates the concentrations of IL-1β, IL-6, and TNF-α (pg/mL) in the sera of Nico (white), UC (blue), UC + Nico (red), UC + Nor (yellow), UC + Coti (light blue), UC + Anaba (green), UC + Myos (purple), and UC + Anata (gray) groups. (**C**,**D**) The *y*-axis shows the concentrations of IL-1β, IL-6, and TNF-α (pg/mL) per gram of colon in Nico (white), UC (blue), UC + Nico (red), UC + Nor (yellow), UC + Coti (light blue), UC + Anaba (green), UC + Myos (purple), and UC + Anata (gray) groups. Data values are presented as the median ± max/min. * *p* < 0.05 (vs. the UC group). Anaba, anabasine; Anata, anatabine; Coti, cotinine; ELISA, enzyme-linked immunosorbent assay; IL-1β, interleukin-1β; IL-6, interleukin-6; Myos, myosmine; Nico, nicotine; Nor, nornicotine; TNF-α, tumor necrosis factor-α; and UC, ulcerative colitis.

IL-1β, IL-6, and TNF-α levels in the serum and colon were measured using ELISA. The serum level of IL-1β was significantly lower in the UC + Nico group than that in the UC group, with no significant differences in the other two cytokines (Figure 8A). Serum IL-1β was decreased in the UC + Nor group when compared with that in the UC group, and no significant changes in the other three cytokines were noted in the other rat groups (Figure 8B). Considering the colon, secretion of IL-1β and IL-6 was notably decreased in the UC + Nico group when compared with that in the UC group (Figure 8C). Considering the other five groups, secretion levels of IL-1β and IL-6 were significantly reduced in the UC + Nor group when compared with those in the UC group (Figure 8D).

### 3.9. Cytokine mRNA Expression in Colon Tissues

**Figure 9:**
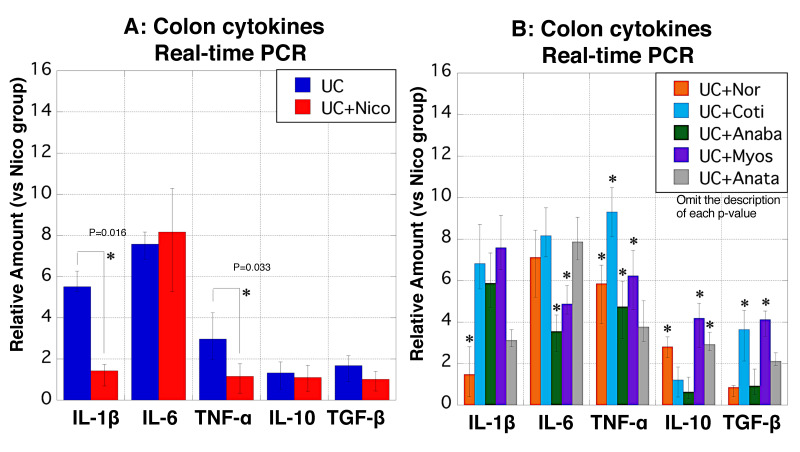
Alterations in colonic mRNA levels of cytokines in rats. (**A**,**B**) Real-time PCR was performed to detect the mRNA expression in rat colon tissues. The *y*-axis indicates the mRNA expression levels of IL-1β, IL-6, TNF-α, IL-10, and TGF-β in the colon tissues of the UC (blue), UC + Nico (red), UC + Nor (yellow), UC + Coti (light blue), UC + Anaba (green), UC + Myos (purple), and UC + Anata (gray) groups, relative to the Nico group. Data values are presented as the median ± max/min. * *p* < 0.05 (vs. the UC group). Anaba, anabasine; Anata, anatabine; Coti, cotinine; IL-1β, interleukin-1β; IL-6, interleukin-6; IL-10, interleukin-10; Myos, myosmine; Nico, nicotine; Nor, nornicotine; TGF-β, transforming growth fctor-β TNF-α, tumor necrosis factor-α; and UC, ulcerative colitis.

We performed real-time PCR to determine the mRNA levels of five cytokines in the colon tissues of all groups. Compared with the UC group, the mRNA levels of IL-1β and TNF-α were significantly downregulated in the UC + Nico group (Figure 9A). The expression levels of IL-1β in the UC + Nor group and IL-6 in the UC + Anaba and UC + Myos groups were significantly decreased, whereas TNF-α expression tended to increase in all five groups (Figure 9B). Regarding anti-inflammatory cytokines, IL-10 expression was significantly upregulated in the three groups, and that of TGF-β was notably increased in the UC + Coti and UC + Myos groups (Figure 9B).

### 3.10. Evaluation of nAChR7 Expression in MΦ

**Figure 10:**
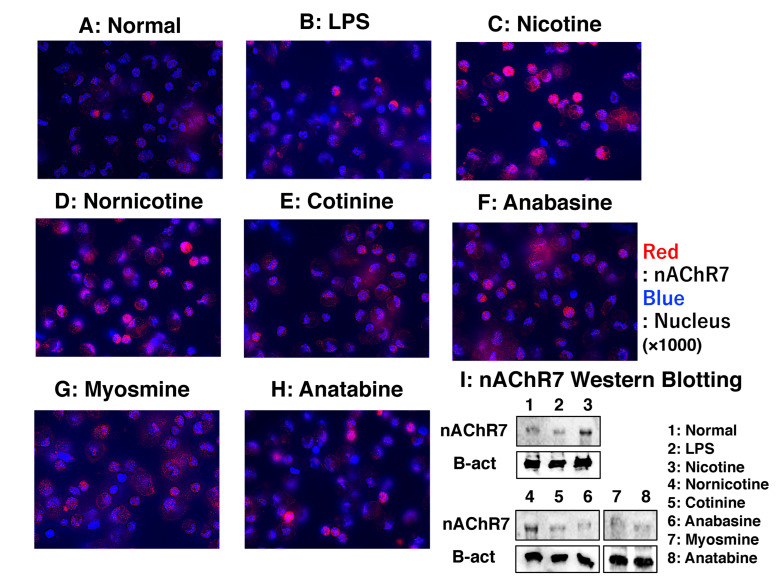
nAChR7 expression in rat peritoneal MΦ at the protein level. (**A**) Results of FICS of nAChR (red fluorescence) in normal rat MΦ (no stimulation). (**B**–**H**) Results of FICS of nAChR in rat MΦ treated with 10 μg/mL LPS, nicotine, nornicotine, cotinine, anabasine, myosmine, and anatabine, respectively. DAPI-stained nuclei exhibit blue fluorescence. All microscopic images were observed using a BIOREVO BZ-9000 microscope (Keyence Co., Ltd., Osaka, Japan). Super-high-power magnification was used for all panels (×1000). (**I**) Western blotting was performed to detect nAChR7 expression in each stimulated MΦ. The upper and lower panels show the expression of nAChR7 and β-actin (an internal control) proteins, respectively. Lane 1 shows the nAChR7 expression in normal rat MΦ (no stimulation). Lanes 2–8 indicate nAChR7 expression in rat MΦ stimulated with 10 μg/mL of LPS, nicotine, nornicotine, cotinine, anabasine, myosmine, and anatabine, respectively. FICS, fluorescent immunochemical staining; LPS, lipopolysaccharide; MΦ, macrophages; and nAChR7, α7 nicotine acetylcholine receptor.

FICS and Western blotting were performed to determine the expression of nAChR7 in MΦ. Based on FICS results, nAChR7 expression was more abundant in nicotine-stimulated MΦ than in other stimulation conditions (Figure 10A–H). Western blotting revealed strong nAChR7 expression in nicotine- or nornicotine-treated MΦ (Figure 10I).

### 3.11. nAChR7 and Cytokine Expression in MΦ

**Figure 11:**
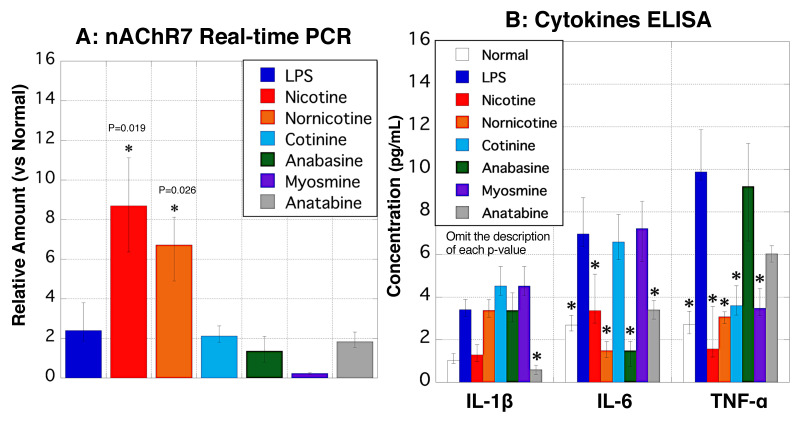
nAChR7 and cytokine expression in rat peritoneal MΦ. (**A**) Real-time PCR was performed to detect nAChR7 expression in MΦ. The *y*-axis shows the mRNA expression levels of nAChR7 in MΦ treated with 10 μg/mL of LPS (blue), nicotine (red), nornicotine (yellow), cotinine (light blue), anabasine (green), myosmine (purple), and anatabine (gray), relative to normal conditions (no stimulation). (**B**) ELISA was performed to measure cytokine levels in rat MΦ. The *y*-axis indicates the concentrations of IL-1β, IL-6, and TNF-α (pg/mL) in normal MΦ (white) or MΦ stimulated with 10 μg/mL of LPS (blue), nicotine (red), nornicotine (yellow), cotinine (light blue), anabasine (green), myosmine (purple), and anatabine (gray). Data values are presented as the median ± max/min. * *p* < 0.05 (vs. LPS treated MΦ). ELISA, enzyme-linked immunosorbent assay; IL-1β, interleukin-1β; IL-6, interleukin-6; MΦ, macrophages; nAChR7, α7 nicotine acetylcholine receptor; and TNF-α, tumor necrosis factor-α.

Real-time PCR and ELISA were performed to evaluate the expression of nAChR7 and cytokines in MΦ. Although the mRNA expression of nAChR7 was not significantly increased in LPS-stimulated MΦ, expression levels were significantly elevated in nicotine- and nornicotine-stimulated MΦ (Figure 11A). The secretion of IL-6 and TNF-α was significantly lower in nicotine- or nornicotine-treated MΦ than that in LPS-stimulated MΦ (Figure 11B).

### 3.12. Changes in Cytokine mRNA Expression in MΦ

**Figure 12:**
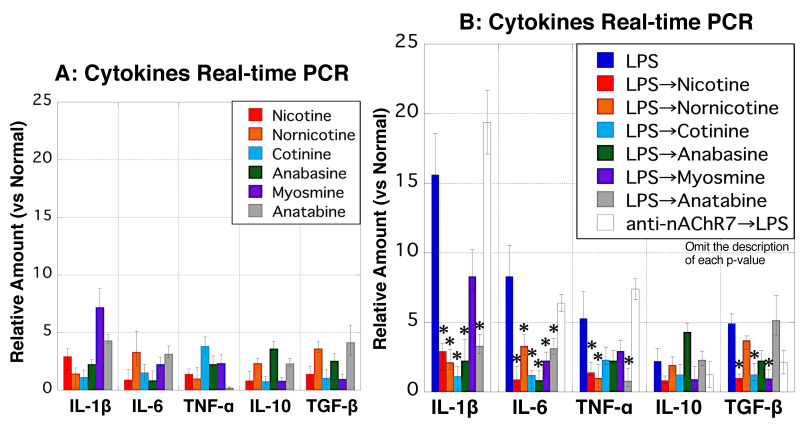
Cytokine mRNA expression in rat peritoneal MΦ. Real-time PCR was conducted to detect cytokines in MΦ. (**A**) The *y*-axis indicates mRNA expression levels of IL-1β, IL-6, TNF-α, IL-10, and TGF-β in MΦ treated with 10 μg/mL of nicotine (red), nornicotine (yellow), cotinine (light blue), anabasine (green), myosmine (purple), and anatabine (gray), relative to normal conditions (no stimulation). (**B**) The *y*-axis indicates the mRNA expression levels of five cytokines in MΦ treated with 10 μg/mL of LPS (blue) or MΦ stimulated with nicotine (red), nornicotine (yellow), cotinine (light blue), anabasine (green), myosmine (purple), and anatabine (gray), after pretreatment with 10 μg/mL LPS. In addition, the condition in which MΦ pretreated with 10 μg/mL anti-nAChR7 and then stimulated with 1.0 mg/mL of LPS was added (white). Data values are presented as the median ± max/min. * *p* < 0.05 (vs. LPS treated MΦ). IL-1β, interleukin-1β; IL-6, interleukin-6; IL-10, interleukin-10; MΦ, macrophages; nAChR7, α7 nicotine acetylcholine receptor; TGF-β, transforming growth factor-β; and TNF-α, tumor necrosis factor-α.

Based on real-time PCR analysis, MΦ stimulation with nicotine or respective structural analogs alone failed to increase the mRNA expression levels of the five examined cytokines (Figure 12A). LPS-stimulated MΦ exhibited elevated mRNA expression levels of the five cytokines, while cytokine production was mostly reduced upon subsequent treatment with nicotine or its structural analogs (Figure 12B). In particular, the mRNA expression of the three inflammatory cytokines was suppressed in nicotine- or nornicotine-treated MΦ after LPS stimulation (Figure 12B). Interestingly, LPS-stimulated MΦ pretreated with anti-nAChR7 displayed increased IL-1β and IL-6 expression (Figure 12B).

## 4. Discussion

In the present study, we examined the pharmacological effects of nicotine and its structural analogs in a rodent model of experimental UC. Initially, we speculated that nicotine-mediated improvements in inflammation observed in human UC were merely a psychological effect of stress relief. Therefore, we performed animal experiments in rats to assess whether nicotine can induce therapeutic benefits against inflammation. Several previous studies have verified the functional role of nicotine in animal models of UC [18,19]. Herein, we focused on substances with chemical structures similar to that of nicotine, predicting that the structural analogs would likely exert anti-inflammatory activity comparable to that of nicotine. Moreover, identifying structural analogs that are less dependent and addictive than nicotine while exhibiting superior pharmacological effects may be valuable. The present study also contributes to identifying the specific molecular structures that underlie the pharmacological effects mediated by nicotine. Herein, we performed a functional comparison between nicotine and its structural analogs, which will provide useful evidence for pursuing related studies worldwide.

In the present study, data regarding changes in rat body weight had substantial implications. The gradual decrease in body weight of rats administered nicotine daily suggests that nicotine might exert a toxic effect or enhance basal metabolism. Furthermore, substances with a closely related chemical structure to nicotine could be associated with strong basal metabolic activity. Although smoking cessation has long been reported to decrease basal metabolic rate and increase body weight [29], we noted that even short-term nicotine administration could lead to apparent weight suppression. In addition, patients with UC tend to lose weight owing to repeated diarrhea and hemorrhage [12], and the accelerated weight loss caused by nicotine is undesirable in UC. Therefore, regardless of the pharmacological effect of nicotine on UC, excessive dosing should be avoided. The colon diameter of rats tends to atrophy with worsening experimental UC. However, the maintenance of colon length in UC rats, especially those treated with nicotine or nornicotine, indicates the potential of these chemicals as UC therapeutic agents. Given that the loss of colonic folds and mucosal epithelium with strong inflammation leads to the shortening of the large intestine, nicotine and nornicotine may act as anti-inflammatory substances. In contrast, the administration of cotinine, which is produced in the liver as a nicotine metabolite, is unlikely to suppress colon shortening in UC. We speculate that once nicotine is metabolized in the body, its pharmacological effects may be substantially lost. According to previous reports, nicotine treatment of neutrophils inhibited free radicals’ production by up to 90.2%, but cotinine treatment of them inhibited free radicals production by only 58.9% [30]. Since nicotine would be superior to cotinine in terms of the free radical’s inhibition, it is likely that the same function was demonstrated in the colon tissue in the experimental colitis of the present study. Furthermore, subtle structural differences between nicotine and cotinine may significantly impact their anti-inflammatory function. We conducted additional experiments to examine the detailed role of these chemicals in UC-mediated inflammation.

Furthermore, in terms of DAI scores, nicotine treatment may exert a better anti-inflammatory effect than the examined analogs. Given that DAI scores increased with body weight loss and considering the weight change results, the score is likely to increase with nicotine administration. However, mild diarrhea was observed in nicotine-treated rats with UC, and the lack of rectal bleeding may have contributed to these results. In nicotine-treated UC rats, H&E-stained colon tissue specimens displayed a preserved mucosal epithelial structure, and the loss of goblet cells seemed to be suppressed. The histological findings also corroborated the pharmacological effect of nicotine. Interestingly, despite nicotine administration in rats with UC, a certain amount of immune cell infiltration was observed in colon tissues, suggesting that nicotine is not actively involved in suppressing immune cell induction or apoptosis. According to previous experiments in which nicotine was administered to mouse models of acute lung injury, nicotine appears to have a particularly potent inhibitory effect on the production of cytokines and chemokines in the inflammatory sites [31]. Therefore, nicotine may suppress the abnormal activity of immune cells or regulate their function to normal. Next, we examined the expression levels and activity of the nicotine-specific receptor, nAChR7. Surprisingly, we revealed poor nAChR7 expression in the colon of WT rats administered nicotine intraperitoneally; however, colonic expression of nAChR7 dramatically increased in nicotine-treated rats with UC. These findings suggest that inflammation triggers the colonic expression of nAChR7 and can impact immune function by promoting nicotine uptake into nAChR7. These findings are mediated via an autocrine activation pathway, suggesting that nicotine uptake by nAChR7 further triggers receptor binding to nicotine. Given the poor nAChR7 expression in UC-induced inflamed colon tissues, administering chemicals such as nicotine, which enhances receptor activity, could effectively treat UC. Tropisetron, a 5-HT_3_ antagonist, can reportedly increase nAChR7 expression in the colon tissues of DSS-treated rats and alleviate colitis [19]. Thus, nAChR7 activators, including nicotine, could be explored as potential therapeutic agents for UC. Upon nicotine or nornicotine administration, IL-1β showed a notably decreased level in the serum and colon of UC rats, with IL-6 also demonstrating a decreasing tendency, mainly in the colon tissues. IL-1β is known to exert various physiological activities. Most importantly, IL-1β promotes the differentiation and proliferation of helper T cells. In particular, IL-1β and IL-6 stimulate helper T cells to differentiate into Th17 cells, which eventually secrete potent cytokines such as IL-17 and TNF-α, resulting in the deterioration of UC [32]. In addition, IL-1β and IL-6 are closely involved in neutrophil induction at inflammatory sites and B lymphocyte proliferation, indicating that nicotine and nornicotine, which suppress these factors, may exert superior effects as immunosuppressive agents. We found that nicotine treatment suppressed the colonic expression of TNF-α mRNA in UC rats; though mRNA levels of anti-inflammatory cytokines, such as IL-10 and TGF-β, increased under some conditions, they failed to significantly impact the pathogenesis of UC. Collectively, it should be noted that nicotine could suppress inflammatory cytokine secretion via nAChR7 activation in colon tissues, thereby alleviating inflammation in UC.

Although the functions of nicotine and its structural analogs have been estimated by analyzing the pathology and immunocompetence of UC model rats, their direct effects on immune cells have not been thoroughly investigated. Herein, we cultured MΦ extracted from the abdominal cavity of rats and examined the regulation of their immune capacity via direct stimulation with nicotine or its structural analogs. Although LPS is known to activate immune cells, LPS-induced MΦ stimulation did not appear to specifically increase nAChR7 levels when compared with those of unstimulated cells. Furthermore, we found that nAChR7 expression was significantly increased in nicotine- or nornicotine-stimulated MΦ when compared with that in cells stimulated with other structural analogs. nAChR7 is abundantly expressed in skeletal muscle and the nervous system and functions as a regulator of synaptic transmission [5,19]. Although the mechanism of indirect MΦ stimulation via nAChR7 expressed in the nervous system is widely established [33], nAChR7 has been recently detected in MΦ, suggesting the direct regulation of immune functions [5]. Given that nAChR7, which does not exhibit high expression in normal MΦ, is upregulated upon stimulation with ligands such as nicotine and nornicotine, it can be suggested that subsequent immunosuppression may be more potent than when cells are stimulated with the other structural analogs. Inflammatory cytokine production was significantly suppressed in nicotine- or nornicotine-stimulated MΦ when compared with that in LPS-stimulated MΦ and seemed almost equivalent to that in unstimulated MΦ. These results indicate that nicotine- or nornicotine-mediated MΦ stimulation does not induce abnormal activity; however, they may act as mediators. It is widely known that nAChR7 exerts anti-inflammatory effects via suppression of NF-κB nuclear translocation and activation of Jak2/STAT3 in neuroinflammation [34]. If this function is also exerted in the colon tissues of patients with UC, nicotine and nornicotine might be effective immunosuppressive agents. Subsequently, we generated data that reinforced this possibility by examining whether MΦ, once activated by LPS, could be restored to a stable state upon treatment with nicotine or its structural analogs. Nicotine and its structural analogs may contribute to the normalization of MΦ by suppressing the mRNA expression of proinflammatory cytokines. Furthermore, it was quite remarkable that treatment of MΦ with anti-nAChR7, followed by LPS stimulation, markedly enhanced expression levels of IL-1β and TNF-α. Anti-nAChR7-induced inhibition of nAChR7 on MΦ resulted in abnormal MΦ activity, indicating that this receptor and its ligands play a substantial role in modulating the innate immune system. Based on our findings, nicotine is the most effective nAChR7 ligand, superior to other structural analogs. In summary, nicotine appeared to bind tightly to nAChR7, inhibit the secretion of inflammatory cytokines, and promote the steady state of MΦ, thereby alleviating inflammation in UC colon tissues. In addition, nicotine activates Nrf2/HO-1, a regulator of oxidative stress, which may contribute to the suppression of UC severity [34]. Nornicotine also exhibits these immunosuppressive and antioxidant functions, but less than that of nicotine, with the other structural analogs exhibiting even weaker activity.

This study aimed to verify whether the structural analogs of nicotine are effective as therapeutic agents for UC; however, nicotine was found to be the most effective pharmacological agent. Although several reports have assessed nicotine previously, reports on the relationship between its structural analogs and UC remain scarce; therefore, the findings of the present study are of considerable value. In particular, our study provides extremely useful evidence, given that the possible involvement of nornicotine in suppressing UC-related inflammation remains poorly explored. Cotinine, a biomarker of tobacco use, is a nicotine metabolite produced in the liver. Herein, we observed that cotinine exerts limited anti-inflammatory effects against UC, and we predict that maintaining the chemical structure of nicotine as feasible would amplify its pharmacological effect. Elevated plasma cotinine has been reported to be associated with an increased risk of developing UC [35], but it seems likely that other toxic substances in cigarettes are responsible for this. Reports demonstrating the efficacy of anabasine and myosmine in treating UC remain scarce, thereby indicating that small differences in their chemical structure from that of nicotine can lead to large gaps in the therapeutic effect against UC. In contrast, oral administration of anatabine alleviated the symptoms of DSS-induced colitis in mice [36], indicating that such plant-derived alkaloids may function as immunosuppressive agents to a certain extent. Evidence regarding the essential role of structural nicotine analogs in UC remains limited, and additional reports can be expected in the future.

In the present study, we identified a potential clinical application for nicotine. The observed pharmacological effect could be attributed to the unique chemical structure of nicotine, and efficient recombination of this structure could lead to the development of a new therapeutic drug targeting UC. However, this study has some limitations. First, given that the experimental period was only 10 days, long-term administration needs to be assessed in future investigations. Considering UC, medication to maintain remission is crucial; therefore, the advantages and disadvantages of administering nicotine or its structural analogs for a prolonged period need to be comprehensively clarified. Second, we only administered a single chemical concentration (1.0 mg/kg), and dosage-based differences in results were not verified. This dosage follows previous papers in which nicotine has shown therapeutic effects on inflammation [25,26], but it may not be the optimal dosage for other nicotinic structural analogs as well. Experimenting with different concentrations of nicotine or its structural analogs may induce differences in the observed results. Third, it is unclear whether intraperitoneal administration, as in the present study, is the best treatment route for UC. Oral, intravenous, and transrectal administration may exert robust pharmacological effects against UC. Given the number of chemicals examined in the present study, it was necessary to conduct a comprehensive analysis; however, this hindered verification across diverse experimental settings, which needs to be explored in future investigations.

## 5. Conclusions

Comparing the short-term outcomes of nicotine and its structural analogs in UC, nicotine administration appears to be the best choice, followed by nornicotine administration. Treatment with nicotine could alleviate experimental UC by activating nAChR7 expression and suppressing the production of proinflammatory cytokines in MΦ. Clinical application of nicotine is currently difficult because of its addictive and dependent effects on humans. Constructing a recombinant form that overcomes these disadvantages would allow the development of UC drugs with reduced toxicity and enhanced pharmacological effects.

## Figures and Tables

**Table 1:** Criteria used for disease activity index (DAI) scoring.

DAI Scores			
Scores	Weight Loss (%)	Stool Consistency	Occult/Gross Bleeding
**0**	None	Normal	Normal
**1**	1–5		
**2**	6–10	Loose stool	Occult bleeding
**3**	11–20		
**4**	>20	Diarrhea	Gross bleeding

**Table 2:** Number of rats per each DAI score at Day 10.

DAI Scores					
	0	1	2	3	4
**Nico**	6	0	0	0	0
**UC**	0	0	2	4	0
**UC + Nico**	0	2	3	1	0
**UC + Nor**	0	2	1	3	0
**UC + Coti**	0	0	2	4	0
**UC + Anaba**	0	0	3	3	0
**UC + Myos**	0	2	1	3	0
**UC + Anata**	0	1	2	3	0

DAI, disease activity index. (*n* = 6 in each rat group).

**Table 3:** Number of rats per each HIS score at Day 10.

HIS Scores									
	0	4	5	6	7	8	9	10	11
**Nico**	6	0	0	0	0	0	0	0	0
**UC**	0	0	0	1	1	2	1	0	1
**UC + Nico**	0	3	2	0	1	0	0	0	0
**UC + Nor**	0	0	0	3	2	1	0	0	0
**UC + Coti**	0	0	0	0	1	2	1	1	1
**UC + Anaba**	0	0	0	0	1	2	1	2	0
**UC + Myos**	0	0	0	1	3	2	0	0	0
**UC + Anata**	0	0	0	0	2	1	2	1	0

HIS, histological. (*n* = 6 in each rat group).

**Table 4:** The colonic mRNA expression levels of nAChR7 measured by real-time PCR.

Relative Amount of Colonic nAChR7 Levels (vs. Nico Group)							
	UC	UC + Nico	UC + Nor	UC + Coti	UC + Anaba	UC + Myos	UC + Anata
**Median**	0.79	6.98	4.67	1.24	1.19	1.14	4.45
**Max**	1.57	7.61	5.24	1.46	1.94	1.55	5.01
**Min**	0.41	6.74	4.24	0.77	0.83	0.87	4.19
***p*-value**		0.022	0.039	0.484	0.528	0.452	0.041
**(vs. UC group)**							

nAChR7, α7 nicotine acetylcholine receptor. (*n* = 6 in each rat group).

## Data Availability

The data presented in this study are available in this article.

## Supplementary Materials

The following supporting information can be downloaded at https://www.mdpi.com/article/10.3390/biomedicines11030922/s1, Figure S1: The content and composition of experimental diets in this study; Figure S2: Raw data of Western blotting in this study; Figure S3: Changes over time in DAI scores for each rat group (mean ± standard deviation).
