# Supplementary material for: Nicotine Exerts a Stronger Immunosuppressive Effect Than Its Structural Analogs and Regulates Experimental Colitis in Rats
Source: Biomedicines. 2023 Mar 16;11(3):922. doi: 10.3390/biomedicines11030922 (PMC10046003; doi:10.3390/biomedicines11030922)
Supplement: Supplementary file 1 [file biomedicines-11-00922-s001.zip › biomedicines-2213135-supplementary.pdf]

The content and composition of experimental diets MF (/100g)

General components		Vitamins		Minerals		Amino acids	
Water (g)	7.9	Vitamin A (IU)	1283	Calcium (g)	1.07	Isoleucine (g)	0.89
Crude protein (g)	23.1	Vitamin D3 (IU)	137	Rin (g)	0.83	leucine (g)	1.74
Crude fat (g)	5.1	Vitamin E (mg)	9.1	Magnesium (g)	0.24	lysine (g)	1.24
Crude ash (g)	5.8	Vitamin K3 (mg)	0.04	Sodium (g)	0.19	Methionine (g)	0.44
Crude fiber (g)	2.8	Vitamin B1 (mg)	2.05	Potassium (g)	0.9	Cystine (g)	0.36
Nitrogen free extract (g)	55.3	Vitamin B2 (mg)	1.1	Iron (mg)	10.6	Phenylalanine (g)	1.04
Calorie (kcal)	359	Vitamin C (mg)	4	Aluminum (mg)	2.1	Tyrosine (g)	0.68
		Vitamin B6 (mg)	0.87	Copper (mg)	0.78	Threonine (g)	0.89
		Vitamin B12 (µg)	5.5	Zinc (mg)	4.89	Tryptophan (g)	0.28
		Inositol (mg)	439	Cobalt (mg)	0.1	Valine (g)	1.08
		Biotin (µg)	27	Manganese (mg)	4.84	Arginine (g)	1.42
		Pantothenic acid (mg)	2.45			Histidine (g)	0.6
		Niacin (mg)	10.61			Alanine (g)	1.2
		Choline (g)	0.18			Aspartic acid (g)	2.14
		Folic acid (mg)	0.17			Glutamic acid (g)	3.99
						Glycine (g)	1.18
						Proline (g)	1.31
						Serine (g)	1.11

Oriental Yeast Co. Ltd. Homepage (Referred to 3/9/2023)
https://www.oyc.co.jp/bio/LAD-equipment/LAD/ingredient.html

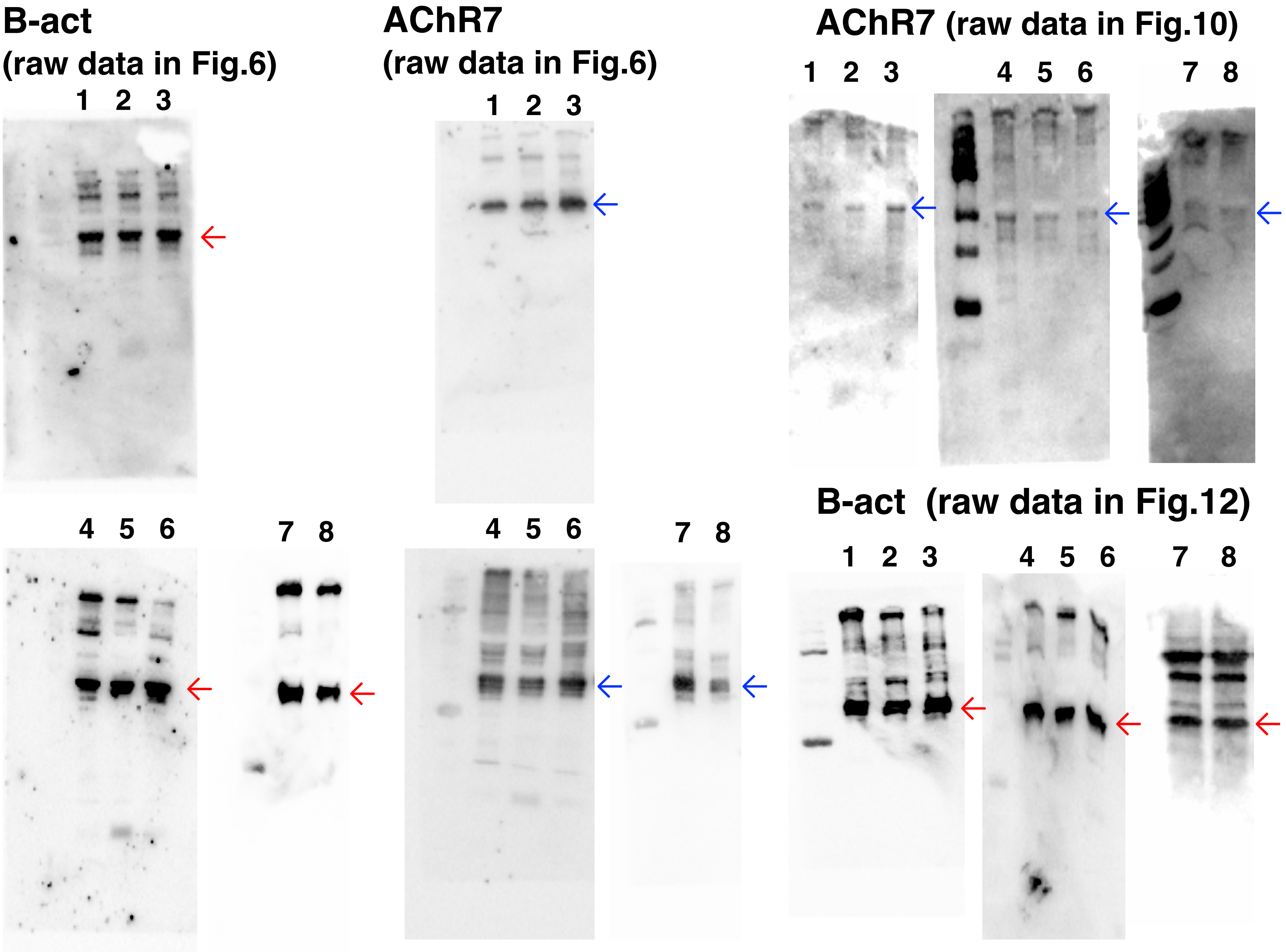

B-act
(raw data in Fig.6)
1 2 3
4 5 6
7 8
AChR7
(raw data in Fig.6)
1 2 3
4 5 6
7 8
AChR7 (raw data in Fig.10)
1 2 3
4 5 6
7 8
B-act (raw data in Fig.12)
1 2 3
4 5 6
7 8

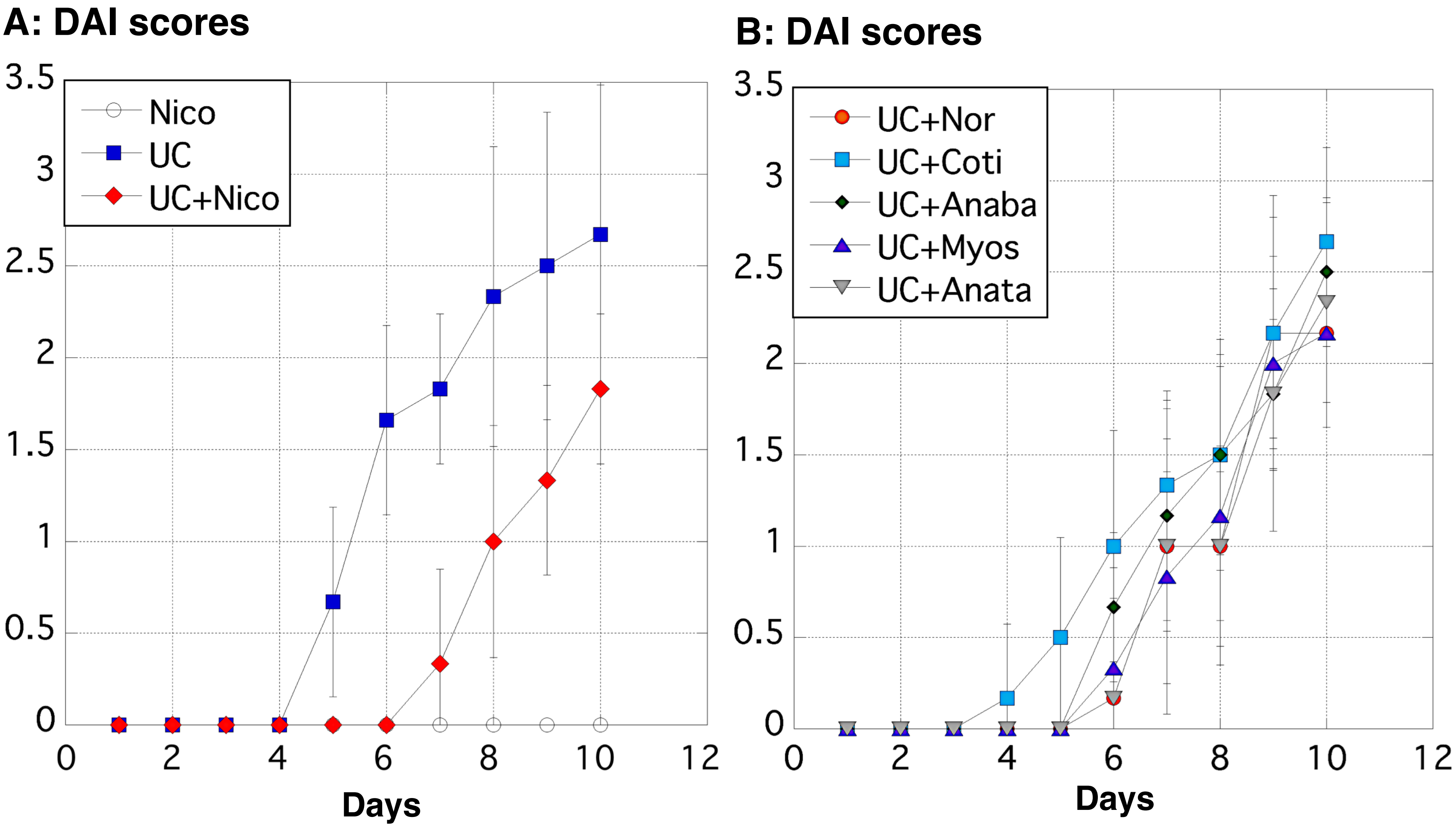
A: DAI scores
Nico
UC
UC+Nico
3.5
3
2.5
2
1.5
1
0.5
0
0
2
4
6
8
10
12
Days
B: DAI scores
UC+Nor
UC+Coti
UC+Anaba
UC+Myos
UC+Anata
3.5
3
2.5
2
1.5
1
0.5
0
0
2
4
6
8
10
12
Days
